# Is Ghana’s pro-poor health insurance scheme really for the poor? Evidence from Northern Ghana

**DOI:** 10.1186/s12913-014-0637-7

**Published:** 2014-12-14

**Authors:** James Akazili, Paul Welaga, Ayaga Bawah, Fabian S Achana, Abraham Oduro, John Koku Awoonor-Williams, John E Williams, Moses Aikins, James F Phillips

**Affiliations:** Navrongo Health Research Centre, PO Box 114, Navrongo, UE/R Ghana; Mailman School of Public Health, Columbia University, New York, USA; Regional Health Directorate, Accra, UE/R Ghana; School of Public Health, University of Ghana, Accra, UE/R Ghana

**Keywords:** National health insurance scheme, Universal health coverage, Pro-poor, Poor, Ghana

## Abstract

**Background:**

Protecting the poor and vulnerable against the cost of unforeseen ill health has become a global concern culminating in the 2005 World Health Assembly resolution urging member states to ensure financial protection to all citizens, especially children and women of reproductive age. Ghana provides financial protection to its citizens through the National Health Insurance Scheme (NHIS). Launched in 2004, its proponents claim that the NHIS is a pro-poor financial commitment that implements the World Health Assembly resolution.

**Methods:**

Using 2011 survey data collected in seven districts in northern Ghana from 5469 women aged 15 to 49 the paper explores the extent to which poor child-bearing age mothers are covered by the NHIS in Ghana’s poorest and most remote region. Factors associated with enrolment into the NHIS are estimated with logistic regression models employing covariates for household relative socio-economic status (SES), location of residence and maternal educational attainment, marital status, age, religion and financial autonomy.

**Results:**

Results from the analysis showed that 33.9 percent of women in the lowest SES quintile compared to 58.3 percent for those in the highest quintile were insured. About 60 percent of respondents were registered. However, only 40 percent had valid insurance cards indicating that over 20 percent of the registered respondents did not have insurance cards. Thus, a fifth of the respondents were women who were registered but unprotected from the burden of health care payments. Results show that the relatively well educated, prosperous, married and Christian respondents were more likely to be insured than other women. Conversely, women living in remote households that were relatively poor or where traditional religion was practised had lower odds of insurance coverage.

**Conclusion:**

The results suggest that the NHIS is yet to achieve its goal of addressing the need of the poor for insurance against health related financial risks. To ultimately attain adequate equitable financial protection for its citizens, achieve universal health coverage in health care financing, and fully implement the World Health Assembly resolution, Ghana must reform enrolment policies in ways that guarantee pre-payment for the most poor and vulnerable households.

## Background

Financial risk protection against the cost of unforeseen ill-health has become a global concern, as evidenced by the 2005 World Health Assembly resolution WHA58.33 which called upon all member states to “plan the transition to universal coverage of their citizens” [1]. In response, even protagonists of user fees have joined the growing consensus that user fees and out-of-pocket payments in general, are *not* appropriate financing mechanisms for health services in developing countries [2,3].

### The global consensus

This consensus has prompted interest in investigating alternative health care financing systems such as tax-based financing, social health insurance and community-based health insurance. Consensus is also emerging that achieving the health Millennium Development Goals (MDGs) by 2015 requires sustainable and equitable long-term health system financing strategies to improve access to health care and potentially to reduce morbidity [4]. However, varying definitions of universal coverage are employed in the policy literature. Universal coverage has been defined by WHO as “access to adequate health care for all at an affordable cost”. An influential definition proposed by McIntyre and Thiede [5] has also defined universal coverage as “a health system that provides all citizens with adequate health care, regardless of their employment status or any other factors”. Kutzin notes that universal coverage is not just access, but that effective coverage implies “necessary health care of good quality” [6] Irrespective of the definition used, two key issues are common to all discourse on this topic: equity in access to quality health care and *financial risk protection*. Achieving universal coverage requires that attention is not only paid to social equity indicators, such as geographical, educational, and cultural barriers, but also to operational constraints to access, such as the quality of care including poor attitude of health staff which discourages the use of health care services particularly by the poor and vulnerable [7,8] but also to financial arrangements. In fact, all commentators agree that universal coverage is a desirable goal in the health system development goal [9]. “The crucial concept in health financing policy towards universal coverage is that of society financial risk pooling” [10]. However, this aspect appears to be ignored in many policy prescriptions for low income countries [3].

Given the failure of user fees [11], increased reliance on pre-payment mechanisms are critical to the achievement of universal access to health services [12]. Currently, there is growing international attention to the design and implementation of health insurance that is addressing the challenges of improving equity, expanding access, and ensuring quality of care provided by health systems in developing countries [12]. Ghana and several other African countries (e.g. Tanzania, Nigeria, Kenya and South Africa) are implementing (or are planning to implement) mandatory health insurance schemes with the aim of ultimately reaching universal coverage.

### The Ghanaian health care financing system

Prior to independence in 1957, user charges were instituted in all public health facilities in Ghana. After independence, health services became free to the public and were financed through general tax revenue. However, sustaining the level of financing required to ensure adequate quality of care and coverage of health services became problematic thereafter.

Following the general economic reforms instituted by the World Bank and the International Monetary Fund (IMF) in 1985, the Ghana Ministry of Health (MOH) introduced significant user fees in public health facilities with the aim of recovering at least 15 percent of recurrent operating costs. Though user fees for clients had existed earlier, the amounts paid were minimal and more of a token. The objective of recovering at least 15 percent of recurrent costs was vigorously pursued and met by Ghana [13]. However, access and utilization studies showed a significant reduction in the use of health services, especially in rural areas, after the introduction of user fees [13,14]. User fees, commonly called the ‘cash and carry’^a^ system in Ghana, undoubtedly contributed to inequitable health service access and utilization between different socio-economic groups and between poor rural and richer urban dwellers [15,16].

In response to these complex problems, the Ministry of Health began to consider the feasibility of health insurance as an alternative to user fees in the late 1980s. By the 1990s, a number of pilot schemes were implemented to test the viability and feasibility of this alternative financing arrangement. Some of the pilot schemes that were set up have led to some increases in utilization and access, promoting equity and efficiency in the areas in which these schemes existed [17]. In addition to these government initiated pilot schemes, a number of community-based pre-payment schemes proliferated and by 2002, there were more than 159 mutual health organizations. Despite this expansion, however, their coverage remained at only about 1 percent (220,000) of the population [17].

### The design of the NHIS

Due to the consistent evidence attesting to the inequities associated with user fees [18], strong political support for pre-payment health care financing came in 2001 when the government announced the introduction of a national health insurance (NHI) scheme to replace ‘cash and carry’ or user fees at the point of service. This policy was translated into legislation in 2003. The NHI coalesces a single policy from multiple schemes, with a district health insurance (DHI) scheme in each of the country’s over 200 districts, private mutual health insurance schemes and private commercial insurance schemes in order to afford all Ghanaians the opportunity to join a health insurance scheme of their choice. The NHI is aimed at providing access to health care for members without having to pay at the point of use and hence improving the affordability of medical care. Adults (outside the formal sector) pay a yearly minimum subscription of US$8.

A National Health Insurance fund (NHIF) has been set up and is financed through a payroll tax contribution, whereby 2.5 percent of the 17.5 percent of formal sector workers’ Social Security Scheme (SSNIT) contributions is directed towards health insurance. This is augmented by a 2.5 percent value added tax on selected goods and services as well as an annual allocation of central government funds. The NHIF transfers funds to each District Health Insurance Scheme (DHIS) based on the number of SSNIT contributors and indigents in the district [19,20]. Existing community-based mutual health insurance schemes continue to function, but each such scheme is now required to be licensed by the National Health Insurance Council (NHIC) and only the NHIF is receiving subsidy from the government. However, private schemes can seek to be incorporated into the district schemes created under the law. The law also makes provision for the licensing, regulation, and operation of private commercial schemes that do not receive government subsidies. A relatively comprehensive NHI benefit package for outpatient and inpatient health services has been assembled that includes maternal care services and covers over 90 percent of the health care burden [20].

Against a background where Out-of-Pocket (OOP) payments currently account for over 45 percent of health care financing in Ghana [3,21], under the NHI the government is seeking to provide quality, accessible, efficient and equitable health services to about 60 percent of all Ghanaians by 2015 and subsequently to obtain universal coverage throughout the country [20]. The current (2012) coverage rate by the scheme has enrolled about 33 percent population, with wide regional variation. Ghana’s ambitious, yet innovative, initiative has elements in common with the health insurance reforms introduced in Thailand [22]. Despite these common features, however, there are widespread concerns about the sustainability of the comprehensive and attractive benefit package covered by the NHI and concerns as well about how equity can be enhanced in ways that introduce a progressive payment regime. As the international community watches with interest Ghana’s giant step toward universal health insurance, there is a need for substantive research aimed at strengthening the design and implementation of the scheme to enhance its progress to universal access through equitable health care financing.

The NHIS is intended to be a pro-poor initiative with a graduated premium payment that is based on socio-economic status, but in reality, premium payments are generally flat due to the practical difficulty in classifying subscribers according to their relative socio-economic status. Planners have attempted to create a scheme for the NHIS that ensures financial protection to the vulnerable including women and children, thereby contributing to the achievement of maternal and child health MDG goals 4 and 5. The analysis that follows explores the extent to which the proposed pro-poor health insurance scheme is actually covering the poor and vulnerable in Ghana’s most impoverished region.

## Methods

### Study site

**Figure 1:**
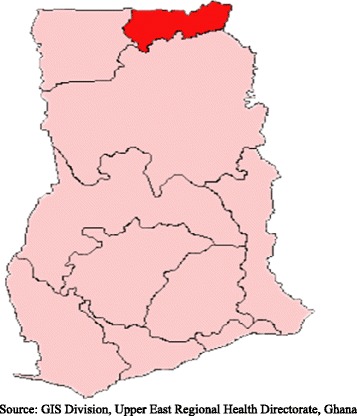
**Map of Ghana showing the Upper East region (study area) shaded.**

Upper East Region is one of the ten regions in the country. It is located in the north-eastern corner of the country between longitude 00 and 10 West and latitudes 100 30″N and 110 N and bordered by Burkina Faso to the north, Togo to the east, to the west by Sissala District in the Upper West Region and to the south by the West-Mamprusi District in the Northern Region (Figure 1). The vegetation of the area is primarily arid savanna grassland with a single growing season. The capital town of the region is Bolgatanga. The total land area is about 8,842 sq km, which translates into 2.7 per cent of the total land area of the country. The major ethnic groups are the Nankani, Bimoba, Bissa, Buli, Frafra, Kantosi, Kassena and Kusasi. The region currently has 11 districts. However, at the time of conducting the baseline survey of the GEHIP project, there were nine districts out of which 7 were study areas of the Ghana Essential Health Intervention Project (GEHIP). The region’s economy is dominated by subsistence agriculture, primarily cattle rearing and the cultivation of cereals like millet, sorghum and rice. The major religions in the area include African animism, Christianity and Islam. With regards to health care, the region has one regional referral hospital and five district hospitals. Apart from government health facilities, there are a few private facilities operated mainly by Christian missionary organizations [23].

### Data

Data for the study come from the GEHIP baseline survey. GEHIP is an intervention study that seeks to test the hypothesis that strengthening district health systems will accelerate progress with MDG 4 and 5. In other words strengthening the key pillars of the health system which include human resource, information management, supply of essential medicines, resource management, leadership and governance and health care financing, could improve MDG 4. The interventions is implemented in three districts (Bongo, Builsa and Garu-Tempane) in the Upper East Region with four other districts (Bolgatanga, Bawku-East, Bawku-West and Talensi-Nabdan) being control areas. Whilst the intervention is being rolled-out by the regional health directorate the baseline and endline surveys which were factored into project were sub-contracted to the Navrongo Health Research Centre. The survey collected data on 5511 women dispersed across 66 randomly selected census enumeration clusters located in both the intervention and non-intervention districts to establish baseline indicators for the study. Doris Duke Charitable Foundation (DDCF) of the USA is the funding agency for GEHIP and the project is technically assisted by Columbia University Mailman School of Public Health in Collaboration with the Ghana Health Service and the University of Ghana, School of Public Health. The project was ethically approved by the Ghana Health Service Ethics committee and Navrongo Health Research Centre Ethics Review board. The baseline survey asked questions to women of child bearing age (15–49 years) after a written consent was obtained. Information was also collected on the National Health Insurance Scheme. Two key questions were asked and these were: *“have you registered as a member of the NHIS”* and a follow-up question to those who had registered: *“Do you have a valid NHIS card for the 2010/2011*”. We took advantage of these questions to assess the coverage of the NHIS by socioeconomic status in the region among women of reproductive age and identified factors affecting enrolment into the scheme since there has been limited comprehensive or wide scale assessment of the NHIS since its inception in 2003/2004.

### Analysis and study variable

Out of 5511 women interviewed in the baseline survey, 42 (0.8 percent) had missing values for health insurance status, and were therefore excluded from the analysis. Basic descriptive statistics and logistic regression analysis were employed in this study. Both bivariate and multiple logistic regression models were used to identify factors affecting enrolment into the scheme. The outcome variable was insurance status categorized as 1 if a respondent was registered into the NHIS and had a valid card and 0 otherwise. The explanatory variables controlled for in the multiple regression models include SES, level of education, place of residence, house size, marital status, age of mother, religion and an autonomy variable. Education was categorized into 3 groups: none versus primary/Junior high versus Secondary or tertiary. Age was categorized into 3 groups: 15–17 yr 18–29 yr olds and 30–49 yr olds. The number of children respondents had was broken down into 3 groups -- 0, 1–4 and 5 or more. Occupation was classified by farmer/trader, civil servant and student. With regards to the level of socioeconomic status, the population was categorized into 5 quintiles, quintile 1 being the poorest and quintile 5 being the most prosperous. The socio-economic status indicator was constructed from the asset data using the Principal Component Analysis [24].

## Results

**Figure 2:**
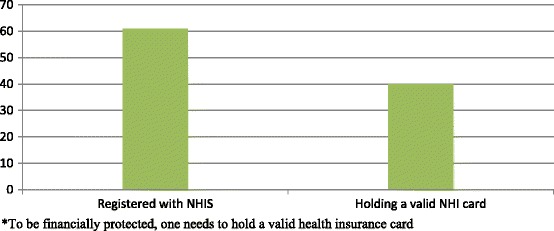
Valid NHIS cards, GEHIP baseline survey 2011*.

Basic descriptive characteristics of insurance status for all 5,469 respondents was limited to location, district, education, age, marital status, SES of household, autonomy, number of children and occupation and are summarized in Table 1. Overall, 60 percent of respondents reported being registered with NHIS and 40 percent reported having a valid NHIS card (Figure 2).

**Table 1:** **Basic characteristics of respondents, GEHIP baseline survey 2011**

**Variable**	**Response options**	**Insured**		**Uninsured**		**p-value**	**Total N = 5469**
		**Number**	**Percent**	**Number**	**Percent**		
**Location**	Rural	1,812	38.1	2,947	61.9		4,759
	Urban	376	53.0	334	47.0	<0.001	710
**District**	Bawku East	449	44.7	556	55.3		1,005
	Bawku West	161	28.1	413	71.9	<0.001	574
	Bolgatanga	252	53.1	223	46.9		475
	Bongo	238	38.3	384	61.7		622
	Builsa	477	51.4	451	48.6		928
	Garu-Tempane	356	30.4	816	69.6		1,172
	Talensi-Nabdan	255	36.8	438	63.2		693
**Education**	None	1,239	37.3	2,085	62.7	<0.001	3,324
	Primary/JSS	766	41.1	1,099	58.9		1,865
	Sec/tertiary	183	65.4	97	34.6		280
**Age**	15–17	431	35.8	774	64.2	<0.001	1,205
	18–29	1,074	44.2	1,356	55.9		2,430
	30–49	683	37.2	1,356	62.8		1,834
**Marital status**	Married	1,519	41.3	2,157	58.7	0.005	3,676
	Not Married	669	37.3	1,124	62.7		1,793
**Socio-economic status of household**	Quintile 1 (poorest)	383	33.9	746	66.1		1,129
	Quintile 2	429	36.9	733	63.1		1,162
	Quintile 3	389	33.3	780	66.7	<0.001	1,169
	Quintile 4	369	38.9	580	61.1		949
	Quintile 5 (richest)	618	58.3	442	41.7		1,060
**Autonomy**	Yes	1,112	39.6	1,699	60.4	0.486	2,811
	No	1,076	40.5	1,582	59.5		2,658
**No of children**	0	560	36.8	962	63.2		1,522
	1–4	1,045	46.1	1,221	53.9	<0.001	2,266
	5+	583	34.7	1,221	65.3		1,681
**Occupation**	Farmer/trader	1,718	39.5	2,629	60.5		4,347
	Civil servant	57	72.2	22	27.9	<0.001	79
	Student	413	39.6	630	60.4		1,043

Majority of the respondents live in rural locations. With regard to place of residence, 53 percent of urban residents were insured while only 38.1 percent of rural residents were insured, indicating that the majority of respondents who live in rural areas where poverty is often perverse, are uninsured (Table 1).

Results are reported (Table 1) for the seven districts including Bawku-West, Bawku-East, Bolgatanga, Bongo, Builsa, Garu-Tempane and Talensi-Nabdan. Whilst Bolgatanga district has the highest percentage (53%) of insured respondents, Garu-Tempane district which is typically the furthest district from the regional capital has the lowest percentage (30%) of insured people.

The frequency of respondents who have insurance with increased monotonically with level of education. For example, while 37 percent of people who had no education were insured, 41.1 percent of those with primary/Junior high were insured. Of those with secondary/tertiary education 65.4 percent were reported to be insured.18 to 29 years old were the highest insured group (44.2%) compared to the 15–17 yr (35.8%) and 30–49 yr (37%) (Table 1). There is evidence of a correlation between marital status and insurance ownership as there seemed to be a higher percentage of uninsured people among unmarried people. Among married people, 41.3 percent were insured compared to 37.3 percent among those not married.

The rate of insurance ownership in quintiles 1–4 were similar with approximately30 percent insured compared to those in the highest quintile (Quintile 5) where 58.3 percent were reported to be insured.

There was minimal difference in insurance status based on the autonomy status of the women. The percentage of respondents with no children (0) was 36.8 percent, those with 1–4 children, 46.1 percent had insurance while 34.7 percent of women with more than 5 children were insured (Table 1). Insurance status varied by occupation as there was a significantly higher rate of insured respondents among the civil servants (72.2 percent) compared to the respondents from households led by farmers, traders or students (40 percent).

Table 2 presents bivariate and multivariate odds ratios estimated with logistic regression analyses. Covariates, including socioeconomic status, educational attainment, place of residence, number of children, marital status, religion and district were all significantly associated with insurance status. Multivariate regression results show that the bivariate results are maintained after controlling for potentially confounding effects of other covariates in the regression.

**Table 2:** **Determinants of NHIS membership among women of reproductive age in the Upper East region, GEHIP baseline survey 2011**

**variable**	**Unadjusted odd ratios**	**95 percent confidence interval**	**Adjusted odd ratios**	**95 percent confidence interval**
**Urban** ^**a**^	1	1	1	1
**Rural**	0.55***	0.47–0.64	0.70***	0.58–0.85
**Bawku East** ^**a**^	1	1	1	1
**Bawku West**	0.48***	0.39–0.60	0.50***	0.39–0.64
**Bolgatanga**	1.40**	1.12–1.74	1.31***	1.02–1.69
**Bongo**	0.77*	0.63–0.94	0.74*	0.57–0.94
**Builsa**	1.31**	1.10–1.57	1.28*	1.02–1.60
**Garu-Tempane**	0.54***	0.45–0.64	0.50***	0.41–0.60
**Talensi-Nabdan**	0.72**	0.59–0.88	0.64***	0.50–0.81
**Quintile (poorest)** ^**a**^	1	1	1	1
**Quintile2**	1.14	0.96–1.35	1.10	0.92–1.32
**Quintile3**	0.97	0.82–1.16	1.06	0.88–1.27
**Quintile4**	1.24*	1.04–1.48	1.35**	1.11–1.63
**Quintile5 (Richest)**	2.72***	2.29–3.24	2.38***	1.96–2.88
**No formal education** ^**a**^	1	1	1	1
**Primary/JSS**	1.17**	1.04–1.32	1.16	0.99–1.36
**Sec/Tertiary**	3.17***	2.46–4.10	1.85***	1.33–2.57
**No child** ^**a**^	1	1	1	1
**1–4 children**	1.47***	1.29–1.68	1.80***	1.40–2.31
**5+ children**	0.91	079–1.05	1.44*	1.07–1.92
**Christianity** ^**a**^	1	1	1	
**Traditional**	0.58***	0.48–0.69	0 .66***	0.54–0.80
**Muslim**	1.08	0.95–1.22	1.23*	1.05–1.45
**No religion**	0.60**	0.44–0.82	0.73	0 .52–1.01
**15–17 years** ^**a**^	1	1	1	1
**18–29 years**	1.42***	1.23–1.64	1.07	0.85–1.35
**30–49 years**	1.07	0.92–1.24	1.06	0.80–1.39
**Not married** ^**a**^	1	1	1	
**Married**	1.18**	1.05–11.33	1.23*	1.01–1.50
**Autonomy** ^**a**^	1	1	1	1
**No autonomy**	1.04	0.93–1.16	1.09	0.96–1.25
**Farmer/trading** ^**a**^	1	1	1	1
**Civil servant**	3.96***	2.42–6.51	1.66	0.92–3.0
**Student**	1.00	0.87–1.15	1.40*	1.08–1.83

Note *p < 0.05 **p < 0.01 ***p < 0.001; ^a^Reference variables.

Compared to urban residents, rural respondents were 30 percent less likely to be insured by the NHIS (OR: 0.70 CI: 0.58–0.85). Considerable variance in district coverage is evident: If Bawku East district is considered as a reference district, the relative odds of NHIS coverage in Bawku West is 50 percent lower, Bongo 30 percent lower, Garu-Tempane 51 percent lower and Talensi-Nabdan 37 percent lower while the relative odds of insurance coverage in Builsa was 28 percent higher and Bolgatanga 31 percent higher (Table 2). Factors explaining these pronounced discrepancies are unknown.

When compared to the poorest Quintile (Quintile 1), the odds of coverage in quintile 2 increases by 10 percent, increases by 3 percent in Quintile 3, by 34 percent in Quintile 4 and doubles in the richest quintile (Quintile 5). However, apart from those in the two highest quintiles where there were statically significant differences from those in the lowest quintile, the rest were not significantly different, suggesting that relative economic standing affects health insurance enrollment among the most prosperous respondents, with no differential effect among the mid-to-lower levels of relative prosperity (Table 2).

Pronounced effects of educational attainment are evident. Respondents who had completed primary or Junior Secondary School had 15 percent increased odds of being enrolled in the NHIS compared to those without any education. On the other hand, those with secondary or tertiary education had 84 percent increased odds of being covered by NHIS compared to those without any education (Table 2).

Reflecting, perhaps the correlation of parity with age, women with 1 to4 children had a 1.8 fold increased odds of being insured compared to those without children while the odds of those with 5 or more children was 1.34 times compared to those without children. Both were statistically significant at 5 percent significance level (Table 2).

The occupational status of mothers also matters when it comes to enrolment in the NHIS. Civil servants had significantly higher odds (OR = 1.66) of being enrolled compared to those engaged in farming or trading. Also, students had significantly higher odds of being registered in the NHIS compared to those in farming/trading.

Religion is associated with insurance coverage. Respondents who professed to be practitioners of traditional religion were associated with a 34 percent decreased odds of being insured compared to respondents who were Christians. Muslims were associated with 1.23fold increased odds of being registered into the NHIS compared to Christians. These were statistically significant at 95 percent confidence level. Married women had a 23 percent increased odds of being insured compared to non-married women (Table 2).

## Discussion

The NHIS is intended to be a pro-poor initiative with a graduated premium payment that is based on socio-economic status [19], but in reality is it really the case? This paper explores the extent to which the proposed pro-poor health insurance scheme is actually covering the poor and vulnerable in Ghana’s most impoverished region. Our results show that respondents from households that are relatively prosperous quintiles were significantly more likely to be insured and therefore more likely to benefit from the NHIS compared to those who are relatively poor. This finding challenges the underlying assumption of the NHIS that the scheme is a pro poor policy that fosters equity in access to care. In this study, 60 percent of the women said they were registered with the NHIS, but only 40 percent of these respondents had valid insurance cards. In our analysis, a participant was considered validly insured if she was registered and also had a valid insurance card.

The Upper East Region of Ghana is the poorest and most remote region of the country [19]. A successful poverty amelioration policy would be expected to have a high coverage in such circumstances. However, not only is the insured coverage rate of 40 percent in the region unacceptably low, pronounced variations in coverage by socioeconomic status are evident. The insurance coverage rate for women in the lowest quintile is only 33.9 percent compared to 58.3 percent among respondents from households in the highest quintile. This finding corroborates with results obtained elsewhere in Ghana and Africa showing that the relatively prosperous are more likely to join the national health insurance than the relatively poor [25]. The reasons for these differences vary and range from financial or economic to social, demographic, political or policy reasons, but in general, results are consistent with the conclusion that equity problems persist, despite nearly a decade of NHIS implementation. Indeed, having no education, having no children, being single, practicing traditional religion, and belonging to a low socioeconomic status household were all characteristics of respondents that were significantly associated with relatively low insurance coverage in the study area.

Moreover, compared to rural dwellers, urban dwellers were significantly found to be more likely to enroll onto the NHIS, perhaps because formal sector workers are required to make compulsorily insurance contributions by payroll deduction, and the formally employed are required to pay a registration fees to collect insurance cards. Since there are more formal sector workers in the urban than in rural areas, urban enrollment rates are higher than rural rates. This finding contrasts with expectations that emerged from an earlier study of respondents in Kassena-Nankana districts of the Upper East Region. Conducted prior to the implementation of the NHIS, respondents were asked about their willingness to join the scheme once it was launched. Rural household members were 3.7 times more likely to claim that they intended to join the NHIS relative to their urban counterparts [26]. Such results suggest that rural urban equity is attainable, and sought after, if the financial burden of enrollment could be removed as an impediment to participation in the scheme.

The level of education of respondents (women of reproductive) also emerged as a determinant of an individual’s NHIS membership status. Individuals with tertiary educational attainment had a 1.8 fold odds of being insured compared to respondents lacking any formal educational exposure. The finding is also consistent with findings among South African women, where respondents who had at least matriculation (secondary) level of education were 2 times more likely to be members of a health insurance scheme than those with lower level of education [27]. Educational attainment generates the potential for individuals to acquire skills, accumulate knowledge, and productively avoid the risk of catastrophic health expenditure [28]. Educational attainment results are closely correlated with occupation. Individuals who are civil servants or government workers had a 1.6 fold odds of being NHIS members compared to those whose occupation was farming.

Respondents who are practitioners of traditional religion were less likely to be insured compared to Christians or Muslims. Muslims were more likely to be insured than Christians. A higher percentage of traditional worshippers do not have formal education so the results on religion are not also too surprising.

The analysis shows that the level of enrollment in the NHIS varies across the seven districts where the survey was conducted. Bolgatanga recorded the highest level of enrollment while Garu-Tempane had the lowest enrollment rates. This results calls for efforts especially in the deprive districts to increase enrolment especially among the poor and vulnerable.

Although age is unassociated with enrolment, married women who are naturally the older ones were more likely to be insured with the NHIS than those who were not married. This finding is consistent with results obtained by Kirigia et al. [27], Harmon and Nolan [29] and Trujillo. Married couples may have a higher demand for health insurance due to the need to protect their children and being more averse to the risk of catastrophic health expenditures than those who are single, separated or divorced [29,30].

## Conclusion

Regression results compiled in this paper provide compelling evidence that the Ghana national health insurance scheme, rather than being pro-poor as was originally formulated, tends instead to differentially benefit the relatively prosperous respondents in a survey conducted in Ghana’s most impoverished region. This finding is robust to controls for possible confounding effects of maternal age and religion and household geographic location. Moreover, respondents who have higher levels of educational attainment and those who are employed in the Civil Service or white colour occupations are more likely to be insured. Urban residents are more likely to be insured than rural counterparts. Since public revenue subsidizes the scheme, the financial underpinning of the scheme is tantamount to differential public investment in the health care needs of the least poor. Charactering the scheme as a “pro-poor” program, at least in Ghana’s Upper East Region, is a misnomer. The results of this analysis therefore attests to the importance of reviewing NHIS pre-payment policies with the goal of enabling the poorest and most vulnerable segments of the population to increase their enrollment levels and benefit from the financial risks associated with health adversity.

### Limitation of the paper

The study covered all districts in the Upper East region except two districts (the Kassena-East and West). These two districts are under the demographic surveillance system undertaken by the Navrongo Health Research centre. As to whether the results of the study would have been different if these two districts were added, one cannot tell but given that the region is generally homogenous; the results might not be different. The study did not also capture certain variables (e.g. education of spouses) which could be important.

## Endnotes

^a^The name was coined from the cost recovery aspects of drugs at public health facilities that required that people make payments before they get drugs to take away.
